# Ethical dilemma in the hospitalization of a transgender patient in Tunisia: balancing safety, gender identity, and institutional constraints

**DOI:** 10.1192/j.eurpsy.2026.10981

**Published:** 2026-07-31

**Authors:** M. Bouchendira, A. Maamri, A. Hajri, A. Karmous, E. Khelifa, H. Zalila

**Affiliations:** Emergencies and consultations, RAZI hospital, Manouba, Tunisia

## Abstract

**Introduction:**

Globally, many hospitals remain gender-segregated, which complicates the care of transgender patients. In Tunisia, the absence of a legal framework and hospital protocols adapted to gender-diverse individuals exposes patients to increased vulnerability.

**Objectives:**

To analyze a clinical case that illustrates the ethical dilemma of hospitalizing a transgender man at high suicidal risk, specifically the decision regarding admission to a male versus a female ward, while examining the tensions between gender identity, patient safety, and institutional constraints.

**Methods:**

We conducted a clinical case analysis based on medical records retrieved from the Emergency and Consultations Department archives. The ethical and clinical reflection, centered on the appropriate placement of the patient, was carried out in collaboration with the hospital ethics committee.

**Results:**

We report the case of a 31-year-old transgender man, undergoing masculinizing hormone therapy, who presented to the emergency department with structured suicidal ideation in the context of a major depressive episode. While he explicitly requested admission to a male ward consistent with his gender identity, institutional safety protocols dictated admission to a female ward. Admission initially exacerbated the patient’s distress and generated uncertainty among staff. This situation raised complex challenges: reconciling the patient’s experienced gender with administrative requirements tied to legal documents, managing the logistical limitations of the service (such as bed availability and the absence of mixed units), and anticipating the reactions of other patients and staff members. To address these issues, the team implemented staff debriefing on transgender and non-binary awareness to promote respectful and inclusive care, and arranged private single-occupancy accommodation to ensure comfort and privacy. Importantly, the patient disclosed delaying consultations in the past due to “Trans broken arm syndrome”, where providers over-focus on trans status at the expense of the presenting complaint. This phenomenon contributes to frustration, stalled treatment, and breaches of confidentiality, underscoring systemic barriers in access to care.

**Conclusions:**

This case underlines the ethical and organizational dilemmas arising from the hospitalization of transgender patients in gender-segregated systems. Beyond individual solutions, the development of inclusive and standardized hospital guidelines is essential in Tunisia to ensure equitable, safe, and dignified care for gender-diverse individuals.

**Disclosure of Interest:**

None Declared

